# Supplementary figures and images for: Malaria prevention in the age of climate change: A community survey in rural Senegal
Source: PLoS One. 2025 Jun 30;20(6):e0313456. doi: 10.1371/journal.pone.0313456 (PMC12208445; doi:10.1371/journal.pone.0313456)

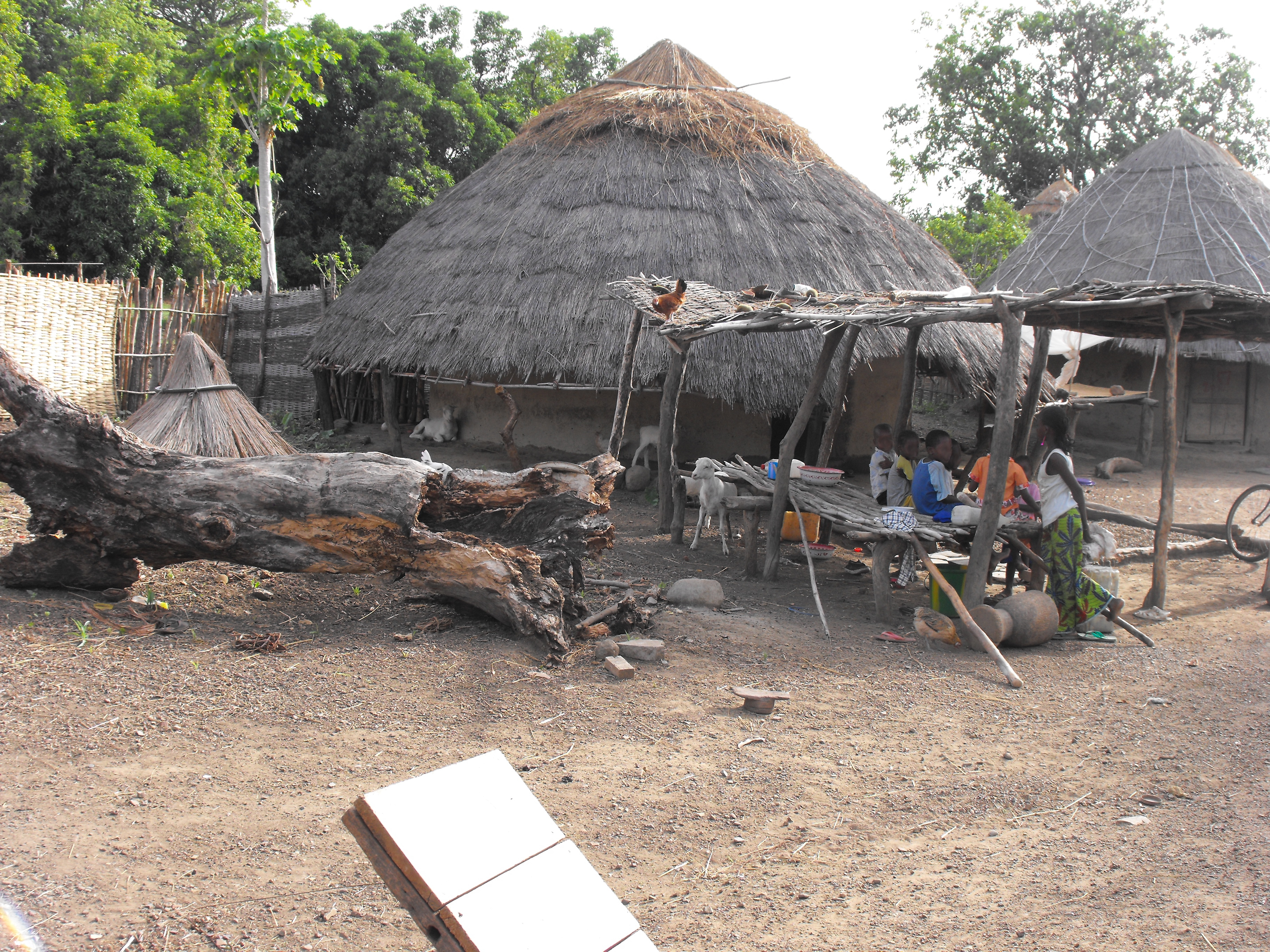

Supplement: S1 Fig — An outdoor shade structure, used during the day for food preparation. In the evening, this structure was a hub for making tea and conversation. For the first half of the night, this structure was regularly used for sleeping. Reprinted from the author’s personal collection, with permission from Andrew Sherman, without copyright restrictions. (ZIP) [file pone.0313456.s001.zip › S1_Fig.tif]

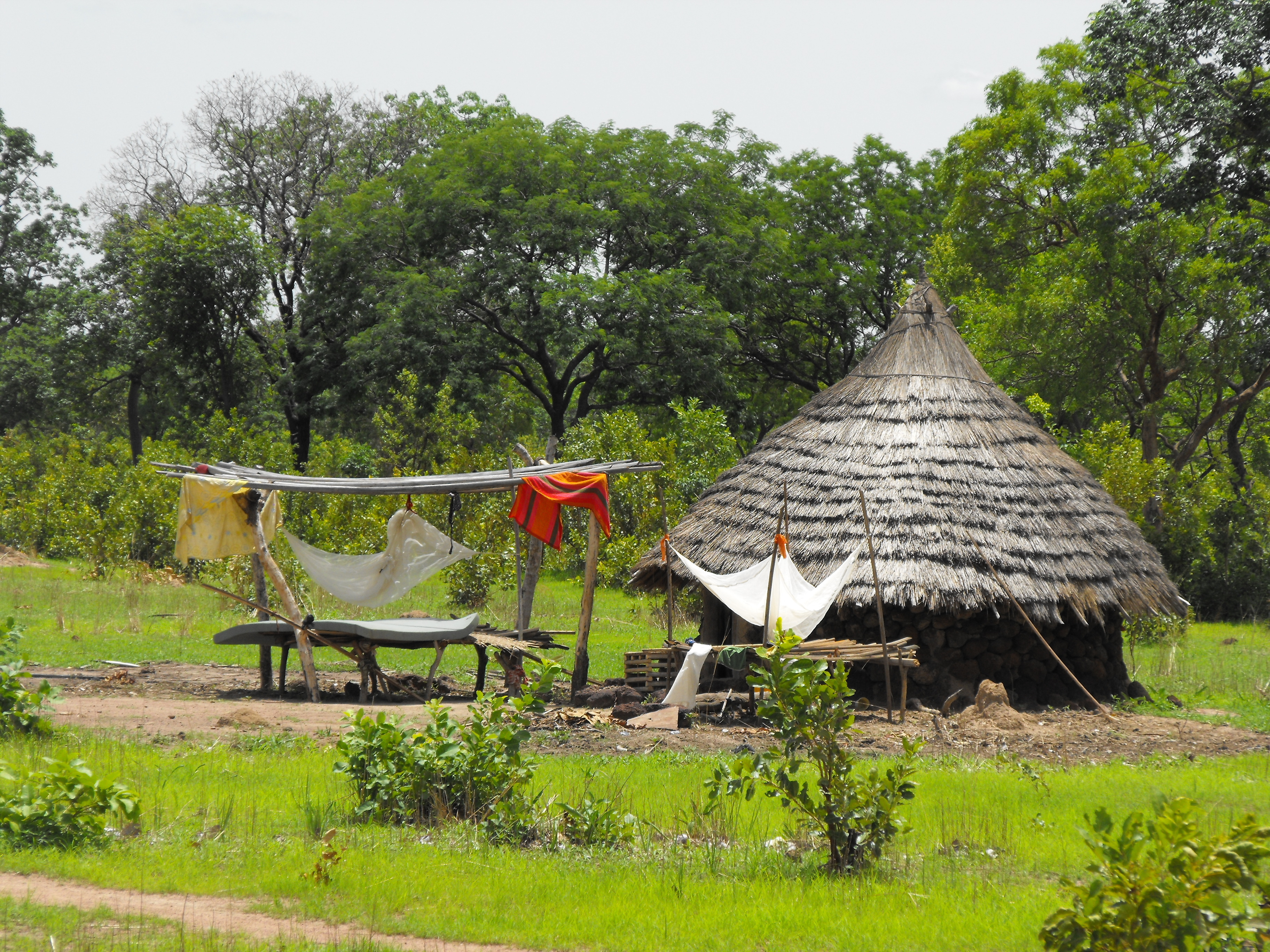

Supplement: S2 Fig — A rare example of outdoor structures with mosquito nets. These elevated sleeping platforms were made with long wood sticks, which typically had sharp ends which catch and tear ITNs. Reprinted from the author’s personal collection, with permission from Andrew Sherman, without copyright restrictions. (ZIP) [file pone.0313456.s002.zip › S2_Fig.tif]

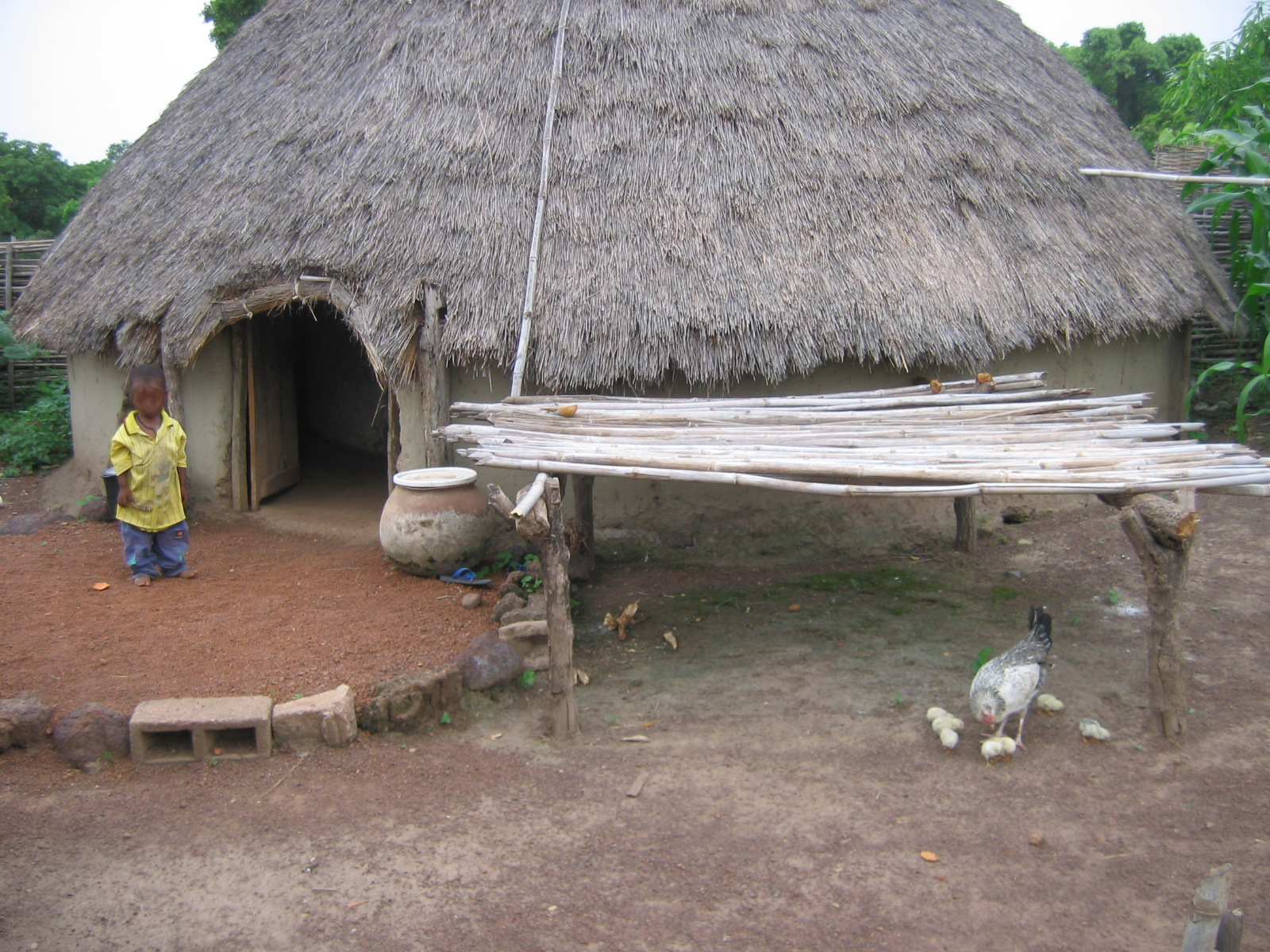

Supplement: S3 Fig — Another example of a typical outdoor structure made with bamboo-like grass. Here, the sharp ends of this material are evident. Reprinted from the author’s personal collection, with permission from Andrew Sherman, without copyright restrictions. (ZIP) [file pone.0313456.s003.zip › S3_Fig.tif]

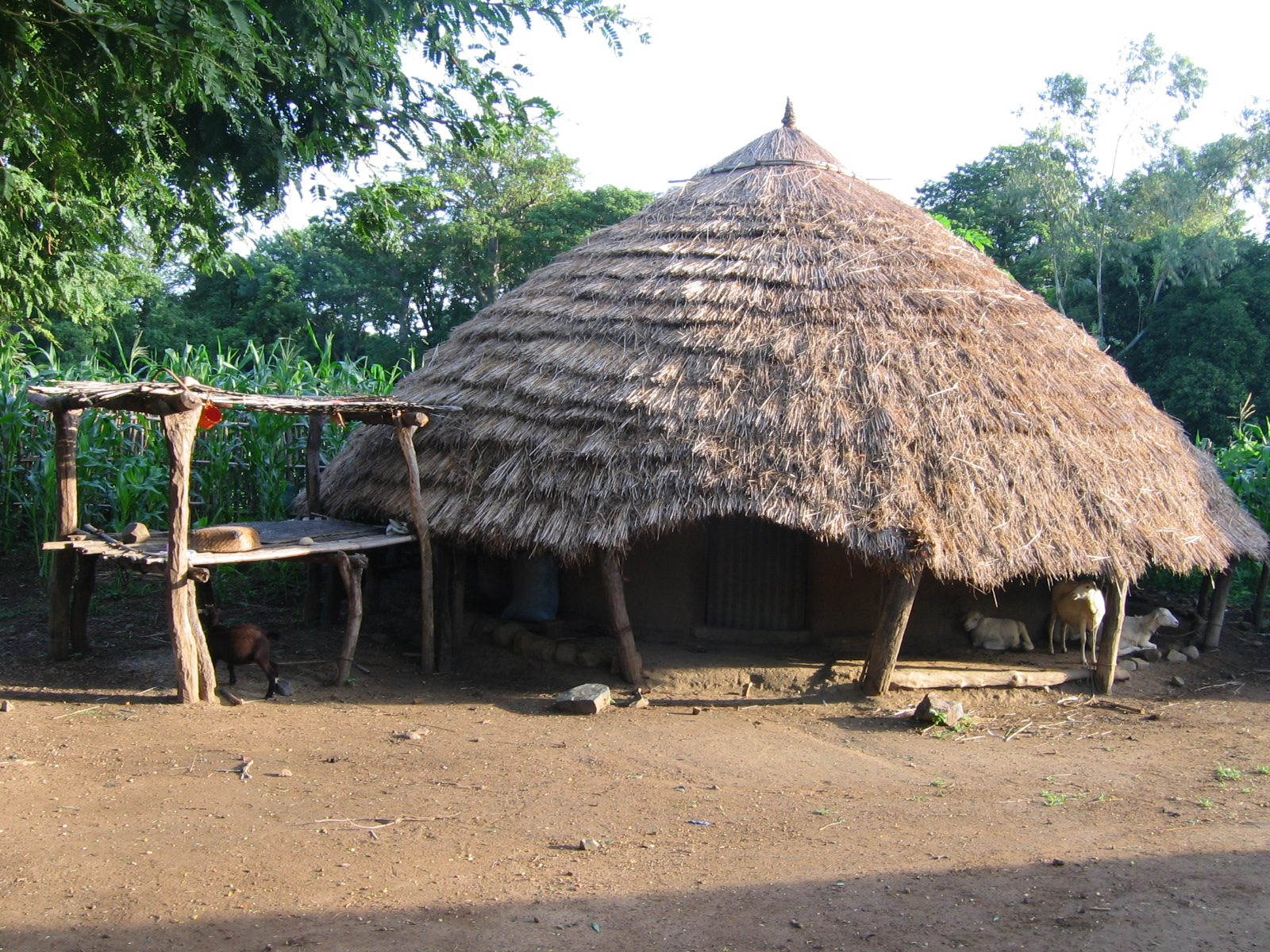

Supplement: S4 Fig — This outdoor structure (left of the hut) was commonly used for drying grain on its top level. Its top level was made from a piece of local wooden fencing, also with sharp edges. Reprinted from the author’s personal collection, with permission from Andrew Sherman, without copyright restrictions. (ZIP) [file pone.0313456.s004.zip › S4_Fig.tif]
