# Supplementary material for: Malaria prevention in the age of climate change: A community survey in rural Senegal
Source: PLoS One. 2025 Jun 30;20(6):e0313456. doi: 10.1371/journal.pone.0313456 (PMC12208445; doi:10.1371/journal.pone.0313456)
Supplement: S1 File — After piloting, the French version of this study’s survey was created. (PDF) [file pone.0313456.s005.pdf]

UIN:

ENQUETE 2012 DE NETLIFE SUR LES MOUSTIQUAIRES AU SENEGAL

Village:	Date:
Nom de l'enquêteur/enquêtrice:	Heure:

Déclaration de consentement:

Bonjour. Je m'appelle ______________________________ et je travaille avec l'université de Rochester aux Etats-Unis. Nous cherchons à connaître comment mieux lutter contre le paludisme. Nous vous prions de bien vouloir participer à cette enquête. Les résultats de cette enquête permettront aux organisations de santé d'améliorer le service rendu. Il faut, en général, entre 10 et 20 minutes pour compléter cette enquête. Les résultats de cette enquête resteront strictement confidentiels.

La participation à cette enquête est libre. Vous pouvez refuser de répondre à une question ou même à toutes les questions. Nous souhaitons votre participation à cette enquête parce que votre avis est important.

Avez-vous besoin de plus d'informations à propos de cette enquête?
Puis-je commencer l'enquête maintenant?

Signature de l'enquêteur: ______________________ Date: ________________

Adapté d'après l'enquête sur les indicateurs du paludisme, Faire reculer le paludisme (RBM) groupe de référence pour la surveillance et l'évaluation, Organisation Mondiale de la Santé, Association humanitaire pour la survie et la protection des enfants du monde (UNICEF), MEASURE DHS, MEASURE Evaluation, et les Centres pour le Contrôle et la Prévention de la Maladie (CDC, U.S.A.), 2005.

Questions pour le chef de ménage (doit avoir plus de 18 ans):

1. Où allez-vous pour les conseils ou les soins quand vous vous sentez malade?

2. Quand vous pensez aux moustiquaires, qu'est-ce que vous aimez?

3. Quand vous pensez aux moustiquaires, qu'est-ce que vous n'aimez pas?

UIN:

Traduction en français:
1.
2.
3.

4. La dernière fois qu'un visiteur d'un autre village est resté chez vous, est-ce qu'il s'est servi d'une moustiquaire? OUI NON

5. La dernière fois que vous êtes resté chez quelqu'un dans un autre village, est-ce que vous vous êtes servi d'une moustiquaire? OUI NON

6. Il y a des gens qui ne se servent pas d'une moustiquaire tous les jours. À votre avis, pourquoi ces gens ne se servent pas tous les jours d'une moustiquaire?

7. Comment est-ce que la moustiquaire pourrait être améliorée?

8. Il y a des gens qui se servent tous les jours d'une moustiquaire. À votre avis, pourquoi ces gens se servent tous les jours d'une moustiquaire?

9. À votre avis, qu'est-ce que les gens du village pourraient faire pour aider la prévention du paludisme? Vous pouvez parler d'autre chose que les moustiquaires.

10. Si il y avait des aides-de-santé spécialisés pour résoudre les problèmes avec les moustiquaires -- et le paludisme en général—est-ce que vous accepteriez d'avoir une visite chez vous pour vous aider?
OUI NON

SI OUI, DEMANDER: A votre avis quelle devrait être la fréquence des visites?

a. Une fois chaque année
b. Deux fois chaque année
c. Quatre fois chaque année
d. Autre: _______________

UIN:

Traduction en français:
4. La dernière fois qu' un visiteur d' un autre village est resté chez vous, est-ce qu' il s'est servi d' une moustiquaire? OUI NON<br><br>5. La dernière fois que vous êtes resté chez quelqu' un dans un autre village, est-ce que vous vous êtes servi d' une moustiquaire? OUI
6.
7.
8.
9.
10. Si il y avait des aides-de-santé spécialisés pour résoudre les problèmes avec les moustiquaires -- et le paludisme en général -- est-ce que vous accepteriez d'avoir une visite chez vous pour vous aider ?<br>OUI NON<br>SI OUI, DEMANDER: A votre avis quelle devrait être la fréquence des visites?<br>a. Une fois chaque année<br>b. Deux fois chaque année<br>c. Quatre fois chaque année<br>d. Autre: ______________

UIN:

ENQUETEUR : MAINTENANT OBSERVER LES MOUSTIQUAIRES

11. Pour chaque habitation: Pouvez-vous me montrer les endroits intérieurs et extérieurs où les membres de votre ménage ont dormi durant la semaine passée, même si c'est juste pour une partie de la nuit?

Endroit pour dormir				Moustiquaire								
No.	Lit intérieur	Sol intérieur	Dehors	Présente?		Accrochée ?		Déchirée ?		Réparation essayée ?		Année obtenue
1				OUI	NON	OUI	NON	OUI	NON	OUI	NON	
2				OUI	NON	OUI	NON	OUI	NON	OUI	NON	
3				OUI	NON	OUI	NON	OUI	NON	OUI	NON	
4				OUI	NON	OUI	NON	OUI	NON	OUI	NON	
5				OUI	NON	OUI	NON	OUI	NON	OUI	NON	
6				OUI	NON	OUI	NON	OUI	NON	OUI	NON	
7				OUI	NON	OUI	NON	OUI	NON	OUI	NON	
8				OUI	NON	OUI	NON	OUI	NON	OUI	NON	
9				OUI	NON	OUI	NON	OUI	NON	OUI	NON	
10				OUI	NON	OUI	NON	OUI	NON	OUI	NON	
11				OUI	NON	OUI	NON	OUI	NON	OUI	NON	
12				OUI	NON	OUI	NON	OUI	NON	OUI	NON	
13				OUI	NON	OUI	NON	OUI	NON	OUI	NON	
14				OUI	NON	OUI	NON	OUI	NON	OUI	NON	
15				OUI	NON	OUI	NON	OUI	NON	OUI	NON	
16				OUI	NON	OUI	NON	OUI	NON	OUI	NON	
17				OUI	NON	OUI	NON	OUI	NON	OUI	NON	
18				OUI	NON	OUI	NON	OUI	NON	OUI	NON	
19				OUI	NON	OUI	NON	OUI	NON	OUI	NON	
20				OUI	NON	OUI	NON	OUI	NON	OUI	NON	
21				OUI	NON	OUI	NON	OUI	NON	OUI	NON	
22				OUI	NON	OUI	NON	OUI	NON	OUI	NON	
23				OUI	NON	OUI	NON	OUI	NON	OUI	NON	
24				OUI	NON	OUI	NON	OUI	NON	OUI	NON	
25				OUI	NON	OUI	NON	OUI	NON	OUI	NON	

12. Est-ce que toutes les moustiquaires que vous avez reçues lors de la dernière distribution sont toujours ici? OUI NON

SI NON, DEMANDER : Qu'est-ce qui est arrivé aux autres moustiquaires ?

UIN:

Continuer ici si nécessaire:

Endroit pour dormir				Moustiquaire				
No.	Lit intérieur	Sol intérieur	Dehors	Présente?	Accrochée ?	Déchirée ?	Réparation essayée ?	Année obtenue
26				OUI NON	OUI NON	OUI NON	OUI NON	
27				OUI NON	OUI NON	OUI NON	OUI NON	
28				OUI NON	OUI NON	OUI NON	OUI NON	
29				OUI NON	OUI NON	OUI NON	OUI NON	
30				OUI NON	OUI NON	OUI NON	OUI NON	
31				OUI NON	OUI NON	OUI NON	OUI NON	
32				OUI NON	OUI NON	OUI NON	OUI NON	
33				OUI NON	OUI NON	OUI NON	OUI NON	
34				OUI NON	OUI NON	OUI NON	OUI NON	
35				OUI NON	OUI NON	OUI NON	OUI NON	
36				OUI NON	OUI NON	OUI NON	OUI NON	
37				OUI NON	OUI NON	OUI NON	OUI NON	
38				OUI NON	OUI NON	OUI NON	OUI NON	
39				OUI NON	OUI NON	OUI NON	OUI NON	
40				OUI NON	OUI NON	OUI NON	OUI NON	
41				OUI NON	OUI NON	OUI NON	OUI NON	
42				OUI NON	OUI NON	OUI NON	OUI NON	
43				OUI NON	OUI NON	OUI NON	OUI NON	
44				OUI NON	OUI NON	OUI NON	OUI NON	
45				OUI NON	OUI NON	OUI NON	OUI NON	
46				OUI NON	OUI NON	OUI NON	OUI NON	
47				OUI NON	OUI NON	OUI NON	OUI NON	
48				OUI NON	OUI NON	OUI NON	OUI NON	
49				OUI NON	OUI NON	OUI NON	OUI NON	
50				OUI NON	OUI NON	OUI NON	OUI NON	

Traduction en français:
12.

13. Quel est le nombre total de personnes dans votre ménage? ________________
Combien de moustiquaires est-ce qu'il faudrait à votre ménage pour que chaque personne puisse dormir sous une moustiquaire (en total, comprenant celles que vous avez déjà)? ______________

14. Si quelqu'un du ménage veut discuter encore a propos des moustiquaires ou du paludisme, s'il vous plait résumer ici. Si possible, noter fidèlement leurs propos.

15. Observations de l'enquêteur/enquêtrice

UIN:

Traduction en français:
13. Quel est le nombre total de personnes dans votre ménage? ______________<br>Combien de moustiquaires est-ce qu'il faudrait à votre ménage pour que chaque personne puisse dormir sous une moustiquaire (en total, comprenant celles que vous avez déjà)? ______________
14.

Tamponner ici. Cachet (du chef de village ou ASC):
Si vous plait, vérifier que la traduction pour toutes les questions est complète:
