# Supplementary material for: Malaria prevention in the age of climate change: A community survey in rural Senegal
Source: PLoS One. 2025 Jun 30;20(6):e0313456. doi: 10.1371/journal.pone.0313456 (PMC12208445; doi:10.1371/journal.pone.0313456)
Supplement: S2 File — The English version of the survey, back translated from French. (PDF) [file pone.0313456.s006.pdf]

English version of survey:

Village:	Date:
Surveyor's name:	Time:

Informed Consent [1]

Hello. My name is ______________________________ and I am working with the University of Rochester in the United States. We are conducting a survey about malaria. We would very much appreciate your participation in this survey. The information you provide will help organizations plan health services. The survey usually takes between 10 and 20 minutes to complete. Whatever information you provide will be kept strictly confidential and will not be shown to other persons.

Participation in this survey is voluntary. You can choose not to answer any individual question or all of the questions. However, we hope that you will participate in this survey since your views are important.

At this time, do you want to ask me anything about the survey?

May I begin the interview now?

Signature of the interviewer: ____________________________ Date: _______________

1. Modified from the Malaria Indicator Survey. Roll Back Malaria Monitoring and Evaluation Reference Group, World Health Organization, United Nations Children's Fund, MEASURE DHS, MEASURE Evaluation, and U.S. Centers for Disease Control and Prevention. 2005.

Questions for head of household (must be over 18 years old):

1. Where do you seek advice or treatment when you feel sick?

2. When you think about mosquito nets, what do you like about them?

3. When you think about mosquito nets, what do you not like about them?

**Translation into French:**
1.
2.
3.

4. The last time a guest from another village visited your household, did they use a net?

Y N

5. The last time you were a guest in another household in another village, did you use a net?

Y N

6. There are people that do not use a mosquito net every day. In your opinion, why do these people not use a net every day?

7. How could the mosquito net be improved?

8. There are some people who use a mosquito net *every day*. In your opinion, why do these people use a net every day?

9. In your opinion, what do you think the people of the village could do to prevent more cases of malaria? You can talk about things other than mosquito nets.

10. If there where health aides who were trained to help solve problems with mosquito nets – and malaria in general—would you be interested in having someone come to the household to help you?

Y N

IF YES, ASK: In your opinion, how often should these people visit your home?

a. Once a year
b. Twice a year
c. Four times a year
d. Other: ________

Translation into French:
4. The last time a guest from another village visited your household, did they use a net? Y N<br><br>5. The last time you were a guest in another household in another village, did you use a net? Y N
6.
7.
8.
9.
10. If there where health aides who were trained to help solve problems with mosquito nets – and malaria in general – would you be interested in having someone come to the household to help you? Y N<br>IF YES, ASK: In your opinion, how often should these people visit your home?<br>a. Once a year<br>b. Twice a year<br>c. Four times a year<br>**d. Other: ________**

SURVEYOR: NOW OBSERVE THE MOSQUITO NETS

11. For each living space: Can you show me the interior and exterior sleeping spaces where members of your household slept during the past week, even if only for part of the night?

	Sleeping space			Mosquito net								
#	Bed, inside	Floor, inside	Outside	Present?		Hung ?		Damaged ?		Repair attempted ?		Year obtained
1				YES	NO	YES	NO	YES	NO	YES	NO	
2				YES	NO.	YES	NO	YES	NO	YES	NO	
3				YES	NO	YES	NO	YES	NO	YES	NO	
4				YES	NO	YES	NO	YES	NO	YES	NO	
5				YES	NO	YES	NO	YES	NO	YES	NO	
6				YES	NO	YES	NO	YES	NO	YES	NO	
7				YES	NO	YES	NO	YES	NO	YES	NO	
8				YES	NO	YES	NO	YES	NO	YES	NO	
9				YES	NO	YES	NO	YES	NO	YES	NO	
10				YES	NO	YES	NO	YES	NO	YES	NO	
11				YES	NO	YES	NO	YES	NO	YES	NO	
12				YES	NO	YES	NO	YES	NO	YES	NO	
13				YES	NO	YES	NO	YES	NO	YES	NO	
14				YES	NO	YES	NO	YES	NO	YES	NO	
15				YES	NO	YES	NO	YES	NO	YES	NO	
16				YES	NO	YES	NO	YES	NO	YES	NO	
17				YES	NO	YES	NO	YES	NO	YES	NO	
18				YES	NO	YES	NO	YES	NO	YES	NO	
19				YES	NO	YES	NO	YES	NO	YES	NO	
20				YES	NO	YES	NO	YES	NO	YES	NO	
21				YES	NO	YES	NO	YES	NO	YES	NO	
22				YES	NO	YES	NO	YES	NO	YES	NO	
23				YES	NO	YES	NO	YES	NO	YES	NO	
24				YES	NO	YES	NO	YES	NO	YES	NO	
25				YES	NO	YES	NO	YES	NO	YES	NO	

12. Are all the mosquito nets you received at the last distribution still here?

YES NO

IF NO, ASK: What happened to the other nets?

Continue here if necessary:

Sleeping space				Mosquito net								
#	Bed, inside	Floor, inside	Outside	Present?		Hung ?		Damaged ?		Repair attempted ?		Year obtained
26				YES	NO	YES	NO	YES	NO	YES	NO	
27				YES	NO	YES	NO	YES	NO	YES	NO	
28				YES	NO	YES	NO	YES	NO	YES	NO	
29				YES	NO	YES	NO	YES	NO	YES	NO	
30				YES	NO	YES	NO	YES	NO	YES	NO	
31				YES	NO	YES	NO	YES	NO	YES	NO	
32				YES	NO	YES	NO	YES	NO	YES	NO	
33				YES	NO	YES	NO	YES	NO	YES	NO	
34				YES	NO	YES	NO	YES	NO	YES	NO	
35				YES	NO	YES	NO	YES	NO	YES	NO	
36				YES	NO	YES	NO	YES	NO	YES	NO	
37				YES	NO	YES	NO	YES	NO	YES	NO	
38				YES	NO	YES	NO	YES	NO	YES	NO	
39				YES	NO	YES	NO	YES	NO	YES	NO	
40				YES	NO	YES	NO	YES	NO	YES	NO	
41				YES	NO	YES	NO	YES	NO	YES	NO	
42				YES	NO	YES	NO	YES	NO	YES	NO	
43				YES	NO	YES	NO	YES	NO	YES	NO	
44				YES	NO	YES	NO	YES	NO	YES	NO	
45				YES	NO	YES	NO	YES	NO	YES	NO	

Translation into French:

12.

13. How many total people are in your household? _______________
How many mosquito nets would your household need so each person could sleep under a mosquito net (total, including the nets that you already have)? _________

14. If a household member wants to further discuss mosquito nets or malaria, please summarize here. If possible, use quotes to document exactly the words the household member uses.

15. Observations of the surveyor

Translation into French:
14.

Stamp here. (Village chief or village health worker):
Please verify that the translation and all of the questions have been completed.
