# Supplementary material for: Malaria prevention in the age of climate change: A community survey in rural Senegal
Source: PLoS One. 2025 Jun 30;20(6):e0313456. doi: 10.1371/journal.pone.0313456 (PMC12208445; doi:10.1371/journal.pone.0313456)
Supplement: S3 File — The local language translation of the study survey. (PDF) [file pone.0313456.s007.pdf]

UIN:

ENQUETE 2012 DE NETLIFE SUR LES MOUSTIQUAIRES AU SENEGAL

Village:	Date:
Nom de l'enquêteur/enquêtrice:	Heure:

Déclaration de consentement:

Oŋ jaaraama. Ko mi wiyette ______________________________ e miɗo liggodade e yimɓe duɗal Rochester New York, aux Etats-Unis. Meɗen daɓutude andude ko ɓuri moyyude lutter fi paludisme. Meɗen ñaagi on moyya tawteɗon laaɓere ko men daɓutude si on jaɓɓi fi bangal cellal ɓeyiditaade ligge wadaade. Nofoti wonnde minitaaji 10 walla 20 fi timinnde o ndetefore. O kesun yaltude o tefore mareteede e nderɓernde.

Ta wede e nde tefore ko hetaare. ɓe wawi salade jabaade e landol walla e landidi fow. Meɗen yidi tawteedon e tefore fi miijo mon no waɗi nafore.

ɗon yidi men ɓeyida na on ha fa mon nde tefore ?

Miɗo wawi fuɗɗaade tefore nden ?

Signature de l'enquêteur: ______________________ Date: ________________

Adapté d'après l'enquête sur les indicateurs du paludisme, Faire reculer le paludisme (RBM) groupe de référence pour la surveillance et l'évaluation, Organisation Mondiale de la Santé, Association humanitaire pour la survie et la protection des enfants du monde (UNICEF), MEASURE DHS, MEASURE Evaluation, et les Centres pour le Contrôle et la Prévention de la Maladie (CDC, U.S.A.), 2005.

Questions pour le chef de ménage (doit avoir plus de 18 ans):

1. Honto yaaton tefiɗagol fi nawnaare mon?

2. Si oŋ miijike fi sanke, ko honduŋ woni on **yidi**?

3. Si oŋ miijike fi sanke, ko honduŋ woni on **yiɗaa**?

UIN:

Traduction en français:
**1.**
**2.**
**3.**

UIN:

4. Sakitoroŋ koɗo ndaaro saare aŋ, o waalike kander sanke?

OUI NON

5. Sakitoroŋ ko wonɗaa ka goɗɗo nder saare, a waalike e sanke?

OUI NON

6. No woodi yimɓe ɓe **waɗata** sanke ñalaaden fof. E miijomon fi honno ɓe **waɗata** sankeejiɗiŋ ñalaaden fof?

7. Ko honno sankeejiɗiŋ wowɗeŋ wade ha ɓuurta?

8. No wooɗi yimɓe ɓe **waɗay** ñalaaden fof sankeejiɗiŋ. E miijomon fi honno yimɓe ɓe **waɗay** sankeejiɗiŋ ñalaaden fof?

9. E miijomon fi honno yimɓe saare wawata wade ha waalabe efeere wonde fi paludisme. Hiɗoŋ waawi haalude e gotuŋ kadi e sankeejiɗiŋ.

10. Si no wooɗi yimɓe wallayɓe fi cellal wakileɓe fi ñawndugol e caɗele fi sankeejiɗiŋ e paludisme, si oŋ jeɓɓay heɓude yimɓe arayɓe ka moŋ fi wallude oŋ?

OUI NON

SI OUI, DEMANDER: E mijamo, honduŋ foti wanude e tawtindirayɓe ndarugol oŋ?

a. Labi goto nder hitanɗe
b. Labi ɗiɗi nder hitanɗe
c. Labi taati nder hitanɗe
d. Honde wonde ? ________________

UIN:

Traduction en français:

4. Sakitoroŋ kodo ndaaro saare aŋ, o waalike kander sanke?

OUI NON

5. Sakitoroŋ ko wondaa ka goddo nder saare, a waalike e sanke?

OUI NON

6.

7.

8.

9.

10. Si no woodi yimɓe wallayɓe fi cellal wakilɓe fi ñawndugol e cadɗe fi sankeejidiŋ e paludisme, si oŋ jeɓɓay hebude yimɓe arayɓe ka moŋ fi wallude oŋ?

OUI NON

SI OUI, DEMANDER: E mijamo, honduŋ foti wanude e tawtindirayɓe ndarugol oŋ?

a. Labi goto nder hitande
b. Labi didi nder hitande
c. Labi taati nder hitande
d. Honde wonde ?

ENQUETEUR : MAINTENANT OBSERVER LES MOUSTIQUAIRES

11. Pour chaque habitation: Pouvez-vous me montrer les endroits intérieurs et extérieurs où les membres de votre ménage ont dormi durant la semaine passée, même si c'est juste pour une partie de la nuit?

**Endroit pour dormir**				**Moustiquaire**				
No.	Lit intérieur	Sol intérieur	Dehors	Présente?	Accrochée ?	Déchirée ?	Réparation essayée ?	Année obtenue
1				OUI NON	OUI NON	OUI NON	OUI NON	
2				OUI NON	OUI NON	OUI NON	OUI NON	
3				OUI NON	OUI NON	OUI NON	OUI NON	
4				OUI NON	OUI NON	OUI NON	OUI NON	
5				OUI NON	OUI NON	OUI NON	OUI NON	
6				OUI NON	OUI NON	OUI NON	OUI NON	
7				OUI NON	OUI NON	OUI NON	OUI NON	
8				OUI NON	OUI NON	OUI NON	OUI NON	
9				OUI NON	OUI NON	OUI NON	OUI NON	
10				OUI NON	OUI NON	OUI NON	OUI NON	
11				OUI NON	OUI NON	OUI NON	OUI NON	
12				OUI NON	OUI NON	OUI NON	OUI NON	
13				OUI NON	OUI NON	OUI NON	OUI NON	
14				OUI NON	OUI NON	OUI NON	OUI NON	
15				OUI NON	OUI NON	OUI NON	OUI NON	
16				OUI NON	OUI NON	OUI NON	OUI NON	
17				OUI NON	OUI NON	OUI NON	OUI NON	
18				OUI NON	OUI NON	OUI NON	OUI NON	
19				OUI NON	OUI NON	OUI NON	OUI NON	
20				OUI NON	OUI NON	OUI NON	OUI NON	
21				OUI NON	OUI NON	OUI NON	OUI NON	
22				OUI NON	OUI NON	OUI NON	OUI NON	
23				OUI NON	OUI NON	OUI NON	OUI NON	
24				OUI NON	OUI NON	OUI NON	OUI NON	
25				OUI NON	OUI NON	OUI NON	OUI NON	

12. Est-ce que sankeejiɗiŋ ko hendi noɗonkoŋ sankitorɗe e sendore deŋ hiɗi ɗoo?

OUI NON

SI NON, DEMANDER : Ko honnuŋ waɗi ɗiya sankeji?

Continuer ici si nécessaire:

Endroit pour dormir				Moustiquaire				
No.	Lit intérieur	Sol intérieur	Dehors	Présente?	Accrochée ?	Déchirée ?	Réparation essayée ?	Année obtenue
26				OUI NON	OUI NON	OUI NON	OUI NON	
27				OUI NON	OUI NON	OUI NON	OUI NON	
28				OUI NON	OUI NON	OUI NON	OUI NON	
29				OUI NON	OUI NON	OUI NON	OUI NON	
30				OUI NON	OUI NON	OUI NON	OUI NON	
31				OUI NON	OUI NON	OUI NON	OUI NON	
32				OUI NON	OUI NON	OUI NON	OUI NON	
33				OUI NON	OUI NON	OUI NON	OUI NON	
34				OUI NON	OUI NON	OUI NON	OUI NON	
35				OUI NON	OUI NON	OUI NON	OUI NON	
36				OUI NON	OUI NON	OUI NON	OUI NON	
37				OUI NON	OUI NON	OUI NON	OUI NON	
38				OUI NON	OUI NON	OUI NON	OUI NON	
39				OUI NON	OUI NON	OUI NON	OUI NON	
40				OUI NON	OUI NON	OUI NON	OUI NON	
41				OUI NON	OUI NON	OUI NON	OUI NON	
42				OUI NON	OUI NON	OUI NON	OUI NON	
43				OUI NON	OUI NON	OUI NON	OUI NON	
44				OUI NON	OUI NON	OUI NON	OUI NON	
45				OUI NON	OUI NON	OUI NON	OUI NON	
46				OUI NON	OUI NON	OUI NON	OUI NON	
47				OUI NON	OUI NON	OUI NON	OUI NON	
48				OUI NON	OUI NON	OUI NON	OUI NON	
49				OUI NON	OUI NON	OUI NON	OUI NON	
50				OUI NON	OUI NON	OUI NON	OUI NON	

**Traduction en français:**
**12.**

13. Ko yimɓe jelo woni kander galle mon? ____________________
Ko sankeji jelu ɗotuɗon heɓude ka galle mon neɗɗo kala wawa lelaade e leiysanke (hara ko joggiɗon koŋ no tawa)? ____________________________

14. Si quelqu'un du ménage veut discuter encore a propos des moustiquaires ou du paludisme, s'il vous plait résumer ici. Si possible, noter fidèlement leurs propos.

15. Observations de l'enquêteur/enquêtrice

UIN:

Traduction en français:

13. Ko yimɓe jelo woni kaŋdes galle maa? ________________________
Ko sankeji jelu foturon hefude ka galle mon neddo kala wawa lelaade e leiysanke (hara ko joggudon koŋ no tawa)? ________________________

14.

Tamponner ici. Cachet (du chef de village ou ASC):
Si vous plait, vérifier que la traduction pour toutes les questions est complète:
