# Supplementary material for: Malaria prevention in the age of climate change: A community survey in rural Senegal
Source: PLoS One. 2025 Jun 30;20(6):e0313456. doi: 10.1371/journal.pone.0313456 (PMC12208445; doi:10.1371/journal.pone.0313456)
Supplement: S1 Checklist — (DOCX) [file pone.0313456.s009.docx]

Inclusivity in global research

PLOS’ policy on inclusivity in global research aims to improve transparency in the reporting of research performed outside of researchers’ own country or community and ensures that PLOS publications reporting global research adhere to high standards for research ethics and authorship. Authors of relevant research articles may be asked to complete the questionnaire below, which outlines ethical, cultural, and scientific considerations specific to inclusivity in global research. This questionnaire may be requested when researchers have travelled to a different country to conduct research, if research uses samples collected in another country, research with Indigenous populations or their lands, or if research is on cultural artefacts. Researchers travelling to another country solely to use laboratory equipment will not normally be required to complete the questionnaire. However, the questionnaire can be requested at the journal’s discretion for any submission – if you have been requested to complete this questionnaire by the PLOS journal you submitted to, please do so.

Please complete the questionnaire below and include this as a Supporting Information file with your manuscript. Note that if your paper is accepted for publication, this checklist will be published with your article in the supporting information files. Please ensure that you reference the checklist in the main body of your manuscript. We suggest adding a subsection ‘Inclusivity in global research’ to your Methods section and adding the following sentence: “Additional information regarding the ethical, cultural, and scientific considerations specific to inclusivity in global research is included in the Supporting Information (SX Checklist)”

The questions have been designed to be applicable to a wide range of study types, and there are subsections for both human subjects research and non-human subjects research. If any of the questions are not relevant to your research please mark them as “N/A” as appropriate.

**Ethical considerations, permits and authorship**

*This section is applicable to all research types.*

Provide details as to who granted permissions and/or consent for the study to take place in the Methods section of your manuscript. This should include the names of all ethics boards, governmental organizations, community leaders or other bodies that provided approval for the study. If individuals provided approval refer to these people by their role or title but do not list their name(s).

Reported on page number: Page 8

If there were any deviations from the study protocol after approval was obtained please provide details of these changes in the Methods section of your manuscript.
Did this study involve local collaborators that are residents of the country where the research was conducted or members of the community studied? If you do not have any authors from said communities, please provide an explanation for this below.

Reported on page number: N/A

This project involved many people in the study area. We met with and discussed this study with each village chief in this area. Each chief helped us gather preliminary census data and verified that each survey was done properly. We collaborated with the group of local *Agents de Santé Communautaires*, who are the village health workers. These community health leaders attended a full day session with us about the details specific to this survey and gave us insights to the proper implementation of the surveys. We piloted this survey in a nearby village and local people gave us feedback on the proper nuances of local language, politeness and acceptance. The surveyors were a diverse group of local people recruited for their representation of the various local cultures, religions and languages. The 100% response to the survey is a testament to the sensitivity and respectfulness of our surveyors and our process.

Most importantly, everything we did benefited from the guidance and approval of the health post nurse of Bandafassi. Mactar Mansaly acted as the primary agent of the Senegalese Ministry of Health and was the provider of health care to all of the population of the surveyed villages. He helped design this study, led the training of surveyors and signed the agreement between this study’s authors and the local health system. All of this was invaluable. Our plan was to discuss data and writing with Nurse Mansaly and to include him as an author. Tragically, he recently passed away. We do pay tribute to him at the end of this manuscript, with the approval of his family.

Despite all of this collaboration, there was no longitudinal partner that contributed sufficiently to meet the criteria of authorship.

Everyone listed as an author should meet PLOS’ criteria for authorship and all individuals who meet these criteria should be included in the author byline, rather than the acknowledgements. For further information please see the journal’s Authorship Policy.

**Human subjects research (e.g. health research, medical research, cross-cultural psychology)**

Did you obtain written informed consent from a representative of the local community or region before the research took place? How did you establish who speaks for the community? Details of written informed consent obtained from study participants should be reported separately in the Methods section of your manuscript.

Yes, written consent was completed. Since the first author of this study lived in these villages for over two years as a Peace Corps Volunteer, we were aware of how leaders represent their people. The village chiefs spoke on behalf of their people. The village health workers spoke to represent the details of maternal and child health. The health post nurse could speak to the health information of the people. We worked with all of these leaders, but we also spent time talking to people who were not leaders, making sure to include the viewpoint of a typical family member in each village. Details regarding consent are included in the Methods section of this manuscript.

How did members of the local community provide input on the aims of the research investigation, its methodology, and its anticipated outcome(s)?

The main goal of the research was to find key information to prevent malaria deaths in children. There is strong local consensus on this goal. The health post nurse, village chiefs, village health workers and non-leader family members all provided input on this study. The health post nurse provided the most information by helping us understand the cultural sensitivity, local resources, and local language nuances to attain the most accurate information and observations.

On a smaller scale, we wanted to know the obstacles to using an available long-lasting insecticide treated mosquito net. Local people helped us with advice on how to ask about these topics, alerting us to any examples about how questions of who sleeps where could be sensitive.

The point of understanding the obstacles was to help find solutions to overcome them. Our group, Netlife, has already used the data from this survey to partner with local villagers in projects to achieve the anticipated outcomes. For example, we sponsored a contest to develop practical tools for using nets outdoors. The winners received prizes, and we have funded local production and dissemination of a hook made from locally available repurposed inner bike tire tubes and wire ([https://youtu.be/OUUquIjP948](https://youtu.be/OUUquIjP948%20) ). We continue to work with the village health worker network, the Ministry of Health Director of the Kédougou Region and the Bandafassi health post staff to support and develop evidence-based projects to prevent malaria deaths in children.

We are not a research entity. We are a tiny, zero-overhead non-profit trying to fight malaria. Publication of this research report will allow us to share our findings about obstacles to net usage with government and non-government organizations, policy makers, scientists and political leaders. We want people in other villages in other regions to benefit from this potentially life-saving information. Our partners in the Kédougou region share this aspiration.

When engaging with the local community, how did you ensure that the informed consent documents and other materials could be understood by local stakeholders?

Verbal consents on all surveys were done in the local language of the family. To prepare for this, we chose our surveyors based on their multilingual skill sets and assigned them to the villages accordingly. Each village chief was also a witness to the survey process to verify that each survey was done properly.

Will the findings of the research be made available in an understandable format to stakeholders in the community where the study was conducted (e.g. via a presentation, summary report, copies of publications, etc.)? Please provide details of how this will be achieved.

Yes. This has already occurred. The non-profit Netlife has discussed the results with the village leaders and health post staff in 2016, 2019, and 2023. This will be an ongoing iterative process as we use these survey data to consult with the local population on the formulation of next interventions. We plan to provide translated copies of this publication to all village chiefs, health post staff and to the leaders at the regional level of the Ministry of Health.

**Non-human subjects research using specimens/ animals collected as part of the study, or those housed in archival collections. Examples include archaeology, paleontology, botany and zoology.**

Did the permission you obtained from a local authority to perform the study include an agreement on access to outputs and benefit sharing? This may include procedures to enable fair distribution of the benefits and resources arising from the research performed. Please include any details of Prior Informed Consent and Benefit Sharing Agreements obtained. These may be required by field-specific regulations, for example the Convention on Biological Diversity (CBD) and the associated Nagoya Protocol.

n/a

If the material used in your study was imported, please A) provide the year it was imported and B) indicate whether permits were obtained to import/export the materials used, C) provide details of any permits obtained. If this information is not available, please indicate this.

n/a

If you used archival specimens, please state how the material used in your study was acquired by the institute it is held in and provide details of any permits obtained for the original excavations/ sample collection. If this information is not available, please indicate this.

n/a

How was the potential cultural significance of the materials collected in your study to local communities considered in your research design? Were Indigenous peoples and/or local researchers and institutions involved with archaeological excavations / collection of specimens? If so, please provide a description of their involvement.

n/a

If your manuscript includes photographs of human remains please indicate whether authors obtained permission from descendants or affiliated cultural communities to do so.

n/a
