# Supplementary material for: Malaria prevention in the age of climate change: A community survey in rural Senegal
Source: PLoS One. 2025 Jun 30;20(6):e0313456. doi: 10.1371/journal.pone.0313456 (PMC12208445; doi:10.1371/journal.pone.0313456)
Supplement: Attachment — Submitted filename: Review report PeerJ.docx [file pone.0313456.s010.docx]

**Review report for PLOS ONE #PONE-D-24-47493 Research Article**

**Journal:** *PLOS ONE*

**Dated:** 3^rd^ January, 2025

**Title:** Malaria prevention in the age of climate change: A community survey in rural Senegal

**Short Title:** Malaria prevention survey in rural Senegal

**Title.**

1. Required minor change if needed. In rural areas is more pronounced or Senegal countryside??

**Introduction**

1. Introduction is very short.
2. Write down about mosquito specie names, and their biology, ecology and dispersion across globe.
3. Which species cause malaria and other associated diseases.
4. Lack of scientific writing in the whole manuscript. Need improvement
5. Aims and objectives are ok.

**Materials and Methods**

1. Line 94 Study population, should be on the start of M&M
2. Lack of required for study
3. Lack of primary & secondary outcomes.
4. Hypothesis.

**Methods**

1. Lack of research question improve more!
2. There are no new technique/methods
3. Outcomes is confused
4. Write down the “Statistical analysis” in the M&M section.

**Results**

1. Lack of demographic presentation.
2. Lack of data presentation
3. Tables & figures need to improve
4. Validity results internal need improve
5. Validity results external require improve

**Discussion**

1. Write down the discrimination statistical and significant studies.
2. Write down the clear findings
3. Need to write comparison with previous research.
4. Discuss strengths of research
5. Write down limitation of research

Conclusion

1. Need reference application of pesticides.
2. Need scientific writing.
3. Global warming reference.
4. Conclusion based on the result so in the manuscript there were no discuss about CO2.

**My suggestion**:

1. Major revision.
2. Check the whole manuscript text wording fonts or formatting.
3. Overall quality of manuscript is good.
4. Need clearer in English writing, grammatical mistakes and preposition etc.
5. Introduction need more improvement
6. Materials and methods needs in the survey questioner would be more interesting in table form
7. Results section the writing of the outcomes is very difficult to understand either would be in table form. There are too may quotation, apostrophe/ punctuation (‘’) marks in the results and discussion section.
8. Discussion,
9. Conclusions need more clear
10. References.
