# Supplementary material for: Malaria prevention in the age of climate change: A community survey in rural Senegal
Source: PLoS One. 2025 Jun 30;20(6):e0313456. doi: 10.1371/journal.pone.0313456 (PMC12208445; doi:10.1371/journal.pone.0313456)
Supplement: Attachment — Submitted filename: Response_to_Reviewers_auresp_2.docx [file pone.0313456.s013.docx]

May 15, 2025

Second Rebuttal Letter for #PONE-D-24-47493

Dear PLOS One Editor,

Overall, we appreciate the feedback from all the reviewers and have used it to substantially improve this manuscript.

Responses to specific comments are listed below. The most recent responses from 4/23/2025 feedback are discussed first. The past responses from 1/29/2025 are also included.

In general, we have clarified how this study’s findings are important for helping to fight malaria and for considering how global warming can have impacts on health.

We understand that this manuscript is not as methodologically sophisticated as global health research can be. Nevertheless, we feel that there is value to this work. This study is based on more than twenty years of connection between the first author, the study team and this population. Dr. Sherman lived in a hut with the people in one of these villages for over two years and has dedicated his life to helping the people of this area fight malaria. The personal interactions of the study team and the people of these villages - at every level - encouraged this community to collaborate with a survey providing the most accurate responses and observations possible.

We believe we have now addressed the general and specific issues raised by the reviewers and await your response.

Sincerely,

Andrew Sherman MD MPH

**JOURNAL REQUIREMENTS AND COMMENTS FROM 4/23/2025**

**Journal Requirements:**

The references for this paper have been reviewed and verified. They are complete and correct. They are styled consistently in the suggested format and compared with other recent high-quality published journal articles in the PLOS One catalog. No referenced articles used have been recently redacted.

**Comments to the Author:**

Questions 1-5 have no current actionable comments or subsequent changes.

Question 6. Review Comments to the Author.

Reviewer #1: I have looked through the authors’ responses to. my concerns and I am fully satisfied with them.Itherefore recommend the paper be accepted.

**Reviewer #4: I appreciate the author to adequately addressing reviewers comments. I have few suggestions that may help improvise the manuscript.**

1. **Please check and rephrase the sentences that started with numbers, which is not in a general practice. For example 42, 45 and 255.**

Lines 40 – 47:

164 of 164 selected households were surveyed, giving a 100% response, representing 21% of this local population. For the 1806 family members, respondents assessed a total need of 1565 nets, implying that each individual in this area needs 0.86 nets (95% CI: 0.77-0.95). Survey responses gave rich, informative responses about mosquito net use. For example: ‘If it's in the room set up properly under the mattress then it will be fine. But if it's outside with the beds that don't have mattresses, then it will deteriorate quickly.’ The main reasons for not using an available net were heat and fragility of the nets. This population had very positive attitudes regarding mosquito nets and appreciated the work of local malaria educators.

Given the recommendation, we changed the first line here to: There was a 100% response rate, with 164 of the 164 selected households surveyed, representing 21% of this local population.

There were no other sentences in this selection that started with a number.

Lines 251-255:

Also, when asked: “Are all the mosquito nets you received at the last distribution still here?” 23.7% said no. When a follow up question asked: “What happened to the other nets?” 69.1% mentioned damage.

Overwashing

When asked what happened to the missing nets, respondents described concerns about how the washing process could damage the nets:

We believe this recommendation here refers to the 23.7% that is used after the quote, which happens to end with a question mark. This makes it appear like 23.7 is the start of a new question. However, in this case, it is the next text after a quotation. So it is not the start of a new sentence, and we will keep this as is.

2. **Paragraph stars from line 118 to 121, the hypothesis may not be required here.**

Lines 118-127:

people [11-12]. This area was selected because it has been a zone of high malaria morbidity and mortality.

Study framework

This study’s research question asked what were the barriers to using an available ITN for people in rural Senegal? A secondary question of this research is how can we quantify the identified barriers and report them in an actionable way that further improves malaria prevention? To address these questions, this study used a framework (Fig 1) used by the Peace Corps specifically to look at malaria control in rural sub-Saharan Africa [16]. This framework combines important aspects of previous models and incorporates a state of sustainability [17]. The Peace Corps framework has potential to guide effective interventions, based on a community’s assessment of barriers to regular ITN use.

After the first submission, we had multiple responses requesting us to be clearer about our research questions and our hypotheses. We wanted to have our hypothesis stated clear and early, and this was put at line 100. We felt that the research question, as a separate component, would fit better next to our framework and design. We wanted to say, here’s the question we want to answer and here’s the framework that makes sense to fulfill this. So we have kept this as is.

3. **In the methodology section, study population description can be concise.**

After our literature review of similar articles in the PLOS One catalog, we noticed that the details here were commonly used (location, population size, timing of rainy season, typical livelihood [urban vs. rural], cultural groups, religion, languages and typical family sizes). Since all of these were seen repeatedly in other journals, we valued this information. It is a sentence or so longer than we sought, but we strongly felt that these elements of describing this population painted the proper picture of these people. And each of these factors has bearing on how this population is affected by malaria and receives public health interventions. This was kept as is.

4. **Lines 139 to 141 can be rephrased or omitted.**

Lines 137-143:

components of behavior, with special attention to barriers of ITN use. The study questions reported here asked about the number of nets needed for everyone in a household to sleep under one, the motivations for people to use their nets, the reasons people may not use nets, ways the nets could be modified to improve use, other ways people believe malaria could be prevented, and possible opportunities for community level interventions to promote the use of ITNs. We also encouraged the surveyors to include observations and relevant comments on the survey. Survey questions are available for review in the appendix (S1-S3 Files).

We will omit the long sentence here listing the main survey questions. We do state the questions later in the results with the quoted responses. And we will have the surveys in the different languages available in the appendix for review.

5. **Lines 196 to 203 can be concise**

Lines 192-202

chiefs of the selected villages, and they approved of this project. Despite this study being exempt, low risk to participants, and having the data analyzed anonymously, we still had each participant give verbal consent before the questionnaire. Each verbal consent was performed in the local language by the surveyor. The script of the verbal consent was modified from the 2005 Malaria Indicator Survey, which was developed by the Monitoring and Evaluation Working Group of Roll Back Malaria [18]. The village chief of each participant served as witness that all aspects of each survey was properly performed, including the verbal consent. To document proper verbal consent, each surveyor signed the survey and each village chief imprinted the survey with the official governmental village stamp. No personal identifiers were recorded. Additional information regarding the ethical, cultural, and scientific considerations specific to inclusivity in global research is included in the Supporting Information (S1 Checklist).

This was a section where the journal editors and reviewers wanted numerous additional details regarding our global health research process. They wanted to know very specific parts of this process, so we wanted to have a lot of information about the step-by-step protocol here. After a few iterations, the result is very transparent and descriptive about our IRB and consent process. The last sentence was required to be in the article verbatim, given the guidelines of PLOS One regarding inclusivity of global research. Due to the recommendations for more information, we would like to leave this section as is.

6. **In conclusion, be precise about take home message.**

This has been a challenge as our original conclusion section was 6 paragraphs long, 411 words and spanned 3 pages! We took it to heart to try to, as one review put it, get it down to 3 sentences.

I’d like to say:

Malaria is still awful in this population.

Why? Because there aren’t enough nets.

Why? Because of the early damage to nets all the outdoor sleeping?

Why? Because it is really really hot and it is getting hotter.

It was hard to say this briefly and eloquently, while including sufficient supporting details. We are happy with the current result that states our main finding in 2 sentences. Then we apply this result to our take home message, which takes 3 sentences. We’d like to keep this current segment as is.

**JOURNAL REQUIREMENTS AND COMMENTS FROM 1/29/2025**

**Journal Requirements:**

1. **Please ensure that your manuscript meets PLOS ONE's style requirements, including those for file naming. The PLOS ONE style templates can be found at**

**https://journals.plos.org/plosone/s/file?id=wjVg/PLOSOne_formatting_sample_main_body.pdf and**

[**https://journals.plos.org/plosone/s/file?id=ba62/PLOSOne_formatting_sample_title_authors_affiliations.pdf**](https://journals.plos.org/plosone/s/file?id=ba62/PLOSOne_formatting_sample_title_authors_affiliations.pdf)

AUTHOR RESPONSE:

The first page (Lines 1-24) were in bold before. These are no longer in bold. The original file names for the supplemental information were without an underscore in the spacing of the text. These were changed so there is an underscore instead of a space for all of these.

2. **In the ethics statement in the Methods, you have specified that verbal consent was obtained. Please provide additional details regarding how this consent was documented and witnessed, and state whether this was approved by the IRB**

AUTHOR RESPONSE:

This study design was evaluated by the University of Rochester Medical Center’s Research Subjects Review Board. It was deemed to be exempt because it does not collect any personal identifiers and posed no significant risk to the participants or their population. We trained local health workers about the specific details to implement this survey. This training was coordinated and led by the Bandafassi Health Post nurse, who is the primary local agent of the Senegalese Ministry of Health. Pictures and details about this training are available upon request. Before any surveys were started, this project’s designers personally visited each of the involved villages. We spoke to each village chief about the details of the project, asking for their permission to perform the surveys. Without any compensation, the village chiefs retallied their village censuses, to update each new birth, death and traveling person within their village since the last health post census. After survey completion, each chief also confirmed that the surveyed participants agreed to the survey and that the survey was done properly. Although the study was exempted by IRB, we still wanted participation to be entirely voluntary. Participants had every opportunity to refuse being surveyed. A script was performed by each surveyor to each head of household regarding the background of the study and that participation was voluntary. Upon receiving verbal consent, the surveyor would continue and complete the survey. After the survey was completed, the village chief verified with the surveyed head of household that the survey was done properly. Each survey has the village chief’s stamp on the last page.

So verbal consent was done by the surveyor, then verified by the chief of the village. We continue to have the scanned files of all the completed surveys, showing the chief’s stamp and signature. These documents will be available for the general public to review in the University of Rochester Research Repository. DOI: 10.60593/ur.d.28533629

3. **Please include a complete copy of PLOS’ questionnaire on inclusivity in global research in your revised manuscript. Our policy for research in this area aims to improve transparency in the reporting of research performed outside of researchers’ own country or community. The policy applies to researchers who have travelled to a different country to conduct research, research with Indigenous populations or their lands, and research on cultural artefacts. The questionnaire can also be requested at the journal’s discretion for any other submissions, even if these conditions are not met. Please find more information on the policy and a link to download a blank copy of the questionnaire here:** [**https://journals.plos.org/plosone/s/best-practices-in-research-reporting**](https://journals.plos.org/plosone/s/best-practices-in-research-reporting)**. Please upload a completed version of your questionnaire as Supporting Information when you resubmit your manuscript.**

AUTHOR RESPONSE:

This form was completed and will be added to the Supporting information files.

4. **Please include your full ethics statement in the ‘Methods’ section of your manuscript file. In your statement, please include the full name of the IRB or ethics committee who approved or waived your study, as well as whether or not you obtained informed written or verbal consent. If consent was waived for your study, please include this information in your statement as well.**

AUTHOR RESPONSE:

Original wording:

Ethical considerations

The University of Rochester Research Subjects Review Board waived this project. The Bandafassi health post administration approved and partnered with this project for the training of the surveyors. We met with all village chiefs of the selected villages, and they approved of this project. Each participant gave verbal consent before the questionnaire. No personal identifiers were recorded.

Revised wording:

Ethical considerations

The University of Rochester Research Subjects Review Board evaluated this project and determined that it met federal and university criteria for exemption. The Bandafassi health post administration approved and partnered with this project for the formulation of aims, objectives and the training of the surveyors. We met with all the village chiefs of the selected villages, and they approved of this project. Each participant gave verbal consent before the questionnaire (Files 1-3). Each verbal consent was performed in the local language by the surveyor. The script of the verbal consent was modified from the 2005 Malaria Indicator Survey [18] I THINK. The village chief of each participant verified that all aspects of each survey were properly performed, including the verbal consent. No personal identifiers were recorded.

Inclusivity in global research

Additional information regarding the ethical, cultural, and scientific considerations specific to inclusivity in global research is included in the Supporting Information (S1 Checklist).

References added:

18. Ndiaye S, Ayad M. Sénégal Enquête Démographique et de Santé 2005. Calverton, Maryland, USA: Centre de Recherche pour le Développement Humain/Sénégal and ORC Macro, 2006

5. **Please provide a complete Data Availability Statement in the submission form, ensuring you include all necessary access information or a reason for why you are unable to make your data freely accessible. If your research concerns only data provided within your submission, please write "All data are in the manuscript and/or supporting information files" as your Data Availability Statement.**

AUTHOR COMMENTS:

Data that were not in the manuscript included scans of the completed surveys and the spreadsheets analyzing the coded responses. These were deposited into the University of Rochester Research Repository. The DOI is 10.60593/ur.d.28533629

6. **We note that Figure 2 in your submission contain [map/satellite] images which may be copyrighted. All PLOS content is published under the Creative Commons Attribution License (CC BY 4.0), which means that the manuscript, images, and Supporting Information files will be freely available online, and any third party is permitted to access, download, copy, distribute, and use these materials in any way, even commercially, with proper attribution. For these reasons, we cannot publish previously copyrighted maps or satellite images created using proprietary data, such as Google software (Google Maps, Street View, and Earth). For more information, see our copyright guidelines:** [**http://journals.plos.org/plosone/s/licenses-and-copyright**](http://journals.plos.org/plosone/s/licenses-and-copyright)**.**

AUTHOR COMMENTS:

We could not confirm copyright status with the non-governmental organization that provided the map data. The authors of this manuscript discussed how much the map added to this research, and we decided to delete the map from the manuscript. If the editors of PLOS One prefer a map to be included, please let us know and we will recreate the map using non-proprietary geographical data.

**Review Comments to the Author and Responses**

**Reviewer 1**

**The authors set out to assess the effect of climate change on malaria prevention using Insecticide treated bed-nets (ITNs) in a high malaria transmission rural area of Senegal. They used a structured questionnaire designed according to the Peace Corps model to conduct a survey in the designated area. They also employed focused interviews to complement the surveys. Although ethical clearance for the survey was obtained at the University of Rochester, USA, the authors did not indicate whether they also obtained ethical clearance from health authorities in Senegal. If they did, they should indicated this in the revised manuscript. If they did not they should explain why this was not done.**

AUTHOR RESPONSE:

This survey study was reviewed and found to be exempt by the University of Rochester Research Subjects Review Board.

We did not obtain formal ethical clearance from health authorities in Senegal. There are several reasons for this.

1. At the time of the survey (2012), local official ethical clearance was not routine practice for such ethnographic public health research projects.
2. No one we worked with in Senegal, or at our home institutional review board, suggested seeking local institutional review board approval.
3. This survey study was low-risk and was indeed found to be exempt by our local IRB. We did not see any potential serious harms for participants in this survey. If anything, we thought that there could be direct benefits to participants to more accurately determining the number of nets needed.
4. Every step of this study involved working with local people, with attention to their health and well-being and also to how they would want to be surveyed.
5. We have long-term connections to key members of the Senegalese Ministry of Health at the village, health post and regional levels. The health post nurse partnered closely in this project to determine the aims and objectives. Nurse Mansaly also helped design the format to train the surveyors specifically for this survey. Our relationships with the village leaders of this area are examples of our ethical dedication to this population.

Nevertheless, we understand the concern being raised by this question. We believe that our approach is proof of our dedication to considering local concerns. We went above and beyond protection of participants to actual community participation in the design and implementation of the study. For example, not only were the surveyors Senegalese, but they were also deliberately chosen to represent diverse languages, religions and cultures present in the region.

We take these matters very seriously. If the reviewer has any specific concerns about any potential ethical lapses in the conduct of this study, we would like to know so we can address those concerns directly.

Our lead author is an adoptive member of a family in one of these villages, with the local name of Ablaye Diallo. After living there for two years, he has returned numerous times over the past twenty years and saved many lives in the region by organizing the distribution of thousands of ITNs. We are not random outsiders exploiting locals for the sake of doing research. If we were starting today, we would obtain the appropriate formal approvals. But our failure to do so at the time is not in any way an indication of our lack of concern for research ethics, subjects’ rights, or the health of the people of the Bandafassi region.

**The main finding of the study is that the use of ITNs has reduced because of increased ambient temperature that makes their use uncomfortable for those surveyed. The authors confirmed that the surveyed population were well informed about malaria and its prevention by the use of ITNs. The rise of temperature mentioned by the authors is not supported by meteorological data from the area studied. Usually such records are kept in francophone African countries. The authors should include supporting data to the claim of climate change and /or variability in the study area.**

AUTHOR RESPONSE:

Additional information was added to the Introduction and Conclusion citing meteorological temperature data in Senegal and how it affects outdoor sleeping.

**I found the following stylistic errors which the authors should correct before the manuscript is published:
1. line 166: Write "All the 164 selected households..."instead of "164 of the164 households..."**

AUTHOR RESPONSE:

Agreed. We changed it to “All 164 selected households “… to be more concise.

**2.line 171: Write, "health care facility/post/centre)'instead of healthcare access...'**

AUTHOR RESPONSE:

The term healthcare access was used because there are two types of healthcare facilities here – a health post and a small hospital. We changed this to say “distance from a local health care facility …” per this recommendation. Thank you.

**3.line 182: Write "What do you think...?" instead of "How do you think..."**

AUTHOR RESPONSE:

We agree that these may be clearer than in the original manuscript text. The phrase is quoted from the survey, after its interpretation into English. Because the survey was worded this way, we chose to report it the exact same way while presenting the responses.

**4 The Discussion should be revised to interpret the results, not to restate them as it currently does. The authors should discuss alternative and/or supplementary methods of malaria prevention such as indoor spraying with insecticides and the use of mosquito traps.**

AUTHOR RESPONSE:

We agree that there is some repetition here and have reworked the Discussion section to be less repetitive and more interpretive. The quotes regarding our process of determining if there was systematic bias were removed from the Discussion, since it seemed like we were continuing to report results in the Discussion section. Additional text was added to let the reader know when we were interpreting results and not just restating or summarizing. Other text was jettisoned to focus on the main results of the study. Segments from the Conclusion were moved to the Discussion section, since they helped interpret the results.

**5.The Conclusion is long and repetitive. Please reduce it to 2-3 sentences highlighting the take home message of the study.**

AUTHOR RESPONSE:

We agree. We reworked the conclusion to be more concise. Some of the text from the Conclusion was moved to the Discussion. Thank you again for all of your recommendations.

**Reviewer 2**

**Title.
1. Required minor change if needed. In rural areas is more pronounced or Senegal countryside??**

AUTHOR RESPONSE:

Although this could be stated either way, most articles in PLOS One use the term “rural.”

**Introduction
1. Introduction is very short.**

AUTHOR RESPONES

Additional information was added to the Introduction.

**2. Write down about mosquito specie names, and their biology, ecology and dispersion across globe.**

**3. Which species cause malaria and other associated diseases.**

AUTHOR RESPONSE:

We considered including more detailed biological information about malaria in this manuscript. Since this study does not add information on how malaria prevention is affected by specific species of malaria parasites or mosquitoes, we chose not to include it. Since we do discuss the climate in the title and in our discussion, we agree that we should include more background on the climate factors in Senegal. We have included this in our revision.

**4. Lack of scientific writing in the whole manuscript. Need improvement**

AUTHOR RESPONSE:

We think that this signifies that this manuscript is not a paper that details the biological details regarding malaria in sub-Saharan Africa. This type of research is extremely important and has been in our literature review for this work. However, this manuscript reports more on the behavioral aspects of mosquito net usage as a public health intervention. We believe the simple descriptive statistics we use are appropriate.

**5. Aims and objectives are ok.**

**Materials and Methods
1. Line 94 Study population, should be on the start of M&M**

AUTHOR RESPONSE:

Study population was moved to the start of Materials and Methods.

**2. Lack of required for study**

AUTHOR RESPONSE:

We are uncertain of this comment and perhaps there is some missing text here. This comment may be asking for better wording in the manuscript to state the reasons this study needed to be done. If so, we have elaborated on this with the hypothesis and outcomes information as discussed below.

**3. Lack of primary & secondary outcomes.
4. Hypothesis.**

AUTHOR RESPONSE:

A change was done in the middle of the introduction. Originally, it was harder to tell what the main hypothesis of the study was. A statement with anticipated outcome was also added. The wording is now:

The primary hypothesis of this study was that people in this region need more than 0.56 nets per person for adequate protection from malaria-bearing mosquitoes. We further hypothesized that net fragility and outdoor sleeping were major factors contributing to the insufficiency of nets.

**Methods
1. Lack of research question improve more!**

AUTHOR RESPONSE:

With the above changes, we added or amended statements regarding aims, hypotheses and outcomes, so they are more clear in the manuscript. A statement discussing the research question was included.

**2. There are no new technique/methods**

AUTHOR RESPONSE:

We agree that we have not introduced new methods of research. Nevertheless, our approach yielded results that differed from current conventional wisdom. While we did use a section of the Malaria Indicator Survey from that time, we added open-ended questions and different observation opportunities for our specially trained surveyors. This is described in the manuscript.

**3. Outcomes is confused**

AUTHOR RESPONSE:

Since this is a survey and not an intervention, we cannot have a clinical outcome that has changed in response to an experimental factor. We are not sure how to respond to this comment. We have generally tried to be clearer throughout the manuscript.

**4. Write down the “Statistical analysis” in the M&M section.**

AUTHOR RESPONSE:

This study does not use advanced statistics. Mainly we make observations and count question responses. For this, we are using only simple descriptive statistics commonly used in surveys.

**Results
1. Lack of demographic presentation**

AUTHOR RESPONSE:

We do not include a typical “Table 1” for this study, because the survey did not request information about the demographics of the participants. Based on our estimates, we surveyed approximately 21% of the entire population of this region. We viewed this as a ample segment of this population, likely accurately representing the responses of the community. We do discuss the languages and religions in the introduction and expect that the various groups of this community are well-represented.

**2. Lack of data presentation**

AUTHOR RESPONSE:

We did our best to present a sample of responses to the survey questions. Other public health malaria surveys often present their data in such a way. It is challenging to create a structure of headings and sub-headings that guides the reader through the main themes and response examples.

**3. Tables & figures need to improve**

AUTHOR RESPONSE:

This may be referring to the fact that this study has no tables or figures to represent the results. Aligning with other similar survey studies, the results are more explained in the language of the respondents. Bar graphs used for these data could oversimplify the types of observations, knowledge and beliefs of the local population. We believe that this method of reporting results best reflects the main findings of this study.

**4. Validity results internal need improve
5. Validity results external require improve**

AUTHOR RESPONSE:

This survey builds on the well-established WHO Malaria Indicator Survey. It was not our intent to create a new survey for general usage. This is a preliminary study by volunteer malaria-fighters seeking to confirm our anecdotal impression about a potentially serious gap in ITN coverage. We believe the questions have face validity. If future researchers would like to build on this work to develop a psychometrically optimized survey for global use, we feel it could be a benefit to this population.

**Discussion**

**1. Write down the discrimination statistical and significant studies.**

AUTHOR RESPONSE:

This study followed the process of discriminant analysis consistent with the common standards of qualitative analysis of survey data. Per the Data analysis section, independent knowledgeable researchers categorized the responses into coded themes. This work was done within a proven framework of peer reviewed past research, building on the modified health belief model.

The references cited in this study were chosen to best reflect the pertinent significant studies, which provide background and context for this research.

**2. Write down the clear findings**

AUTHOR RESPONSE:

The results and discussion have been edited and streamlined to more concisely and clearly state the findings of this study.

**3. Need to write comparison with previous research.**

AUTHOR RESPONSE:

We have added and/or clarified information in the Discussion to address this comment. For example:

Prior to this survey, few studies had specifically investigated outdoor sleeping as a barrier to ITN use. Studies conducted near Lake Victoria and in Burkina Faso found that outdoor sleeping was infrequent [24,25]. In contrast, we found outdoor sleeping to be extremely prevalent, consistent with reports from Niger and Iran [26,27]. Heat has been found to be a significant barrier to ITN use, and causes this study population to regularly sleep outside [28-31].

24. Toé LP, Skovmand O, Dabiré KR, Diabaté A, Diallo Y, Guiguemdé TR, et al. Decreased motivation in the use of insecticide-treated nets in a malaria endemic area in Burkina Faso. Malar J. 2009; 8:175. pmid:19640290

25. Iwashita H, Dida G, Futami K, Sonye G, Kaneko S, Horio M, et al. Sleeping arrangement and house structure affect bed net use in villages along Lake Victoria. Malar J. 2010; 9(1):1–7. pmid:20569459

26. Galvin KT, Petford N, Ajose F, Davies D. An exploratory qualitative study on perceptions about mosquito bed nets in the Niger Delta: what are the barriers to sustained use? J Multidiscip Healthc. 2011; 4:73–83. pmid:21544249

27. Basseri HR, Abai MR, Raeisi A, Shahandeh K. Community sleeping pattern and anopheline biting in southeastern Iran: a country earmarked for malaria elimination. Am J Trop Med Hyg. 2012; 87(3):499-503. pmid:22826491

28. Beer N, Ali AS, Eskilsson H, Jansson A, Abdul-Kadir FM, Rotllant-Estelrich G, et al. A qualitative study on caretakers’ perceived need of bed-nets after reduced malaria transmission in Zanzibar, Tanzania. BMC Public Health. 2012; 12:606. pmid:22863188

29. Pulford J, Hetzel MW, Bryant M, Siba PM, Mueller I. Reported reasons for not using a mosquito net when one is available: a review of the published literature. Malar J. 2011; 10:83. pmid:21477376

30. Ahorlu CS, Adongo P, Koenker H, Zigirumugabe S, Sika-Bright S, Koka E, et al. Understanding the gap between access and use: A qualitative study on barriers and facilitators to insecticide-treated net use in Ghana. Malar J. 2019; 18:417. pmid:31831004

31. Rek J, Musiime A, Zedi M, Otto G, Kyagamba P, Asiimwe Rwatooro J, et al. Non-adherence to long-lasting insecticide treated bednet use following successful malaria control in Tororo, Uganda. PloS one. 2020; 15(12):e0243303. pmid:33270743

**4. Discuss strengths of research**

AUTHOR RESPONSE:

The second paragraph of the Discussion now better highlights strengths of the study.

**5. Write down limitation of research**

AUTHOR RESPONSES:

The limitations of this research are discussed in the Discussion section, paragraphs 3 and 4.

**Conclusion
1. Need reference application of pesticides.**

AUTHOR RESPONSE:

A paragraph discussing other malaria prevention methods was added to the manuscript, citing several papers regarding Indoor Residual Spraying and other outdoor insecticide treated materials.

**2. Need scientific writing.**

AUTHOR RESPONSE:

Sections of this manuscript were revised and reorganized to more clearly state the aims, goals, hypotheses, and conclusions of this research. Additional requested details were added to the Introduction and the Materials and Methods. The Results, Discussion and Conclusions sections had major revisions to more concisely convey the ideas of this research. Sections were also reorganized to reflect a more logical order of scientific process going from Results to Discussion to Conclusions.

**3. Global warming reference.**

AUTHOR RESPONSE:

A global warming reference was added to the manuscript.

3. World Bank Group: Senegal. Climate Change Overview. Country Summary. [Cited 13 March 2025]. Climate Change Knowledge Portal For Development Practitioners and Policy Makers [Internet]. Available from: https://climateknowledgeportal.worldbank.org/country/senegal

**4. Conclusion based on the result so in the manuscript there were no discuss about CO2.**

AUTHOR RESPONSE:

We did take this sentence out.

**My suggestion:
1. Major revision.**

AUTHOR RESPONSE:

Thank you for all of these recommendations and suggestions, and indeed they have been used for a major revision of this manuscript.

**2. Check the whole manuscript text wording fonts or formatting.**

AUTHOR RESPONSE:

This was verified. Font type/size, bolding and indenting are consistent with PLOS One recommendations. (<http://bit.ly/4kHbNAK>)

**3. Overall quality of manuscript is good.
4. Need clearer in English writing, grammatical mistakes and preposition etc.**

AUTHOR RESPONSE:

The text has been reviewed by the authors and other colleagues. Sentence structure, paragraph structure and grammar have been improved if not perfected.

**5. Introduction need more improvement**

AUTHOR RESPONSE:

The Introduction was expanded to clarify the hypothesis. Additional pertinent scientific information regarding meteorologic data and malaria rates has been added.

**6. Materials and methods needs in the survey questioner would be more interesting in table form**

AUTHOR RESPONSE:

We think that for this kind of survey it is preferable to provide quotes of answers and to describe them, rather than to tabulate types of answers.

**7. Results section the writing of the outcomes is very difficult to understand either would be in table form. There are too may quotation, apostrophe/ punctuation (‘’) marks in the results and discussion section.**

AUTHOR RESPONSE:

We did our best to present a sample of responses to the survey questions. Other public health malaria surveys often present their data in this way. We have reduced the number of quoted responses to make it less overwhelming and more concise.

**8. Discussion,
9. Conclusions need more clear**

AUTHOR RESPONSE:

We revised the Discussion and the Conclusion sections to improve the flow.

**10. References.**

AUTHOR RESPONSE:

With the major reorganization of the manuscript, additional references were added.

**Reviewer 3**

**In my humble opinion, the prepared manuscript is decent and highlights one of the important concerns in public health, especially for Africa. However, the manuscript falls short on many aspects. These are as below:**

**1. The authors have documented that there had been reductions in malaria cases and deaths between the periods of 2010-2015; the estimate have now increased in 2025. The conductance of this research was in 2012, a period where one would expect malaria cases to decrease. Important questions that arise what was the prevalence of malaria then and what is now? How many died then and how many now? What were the factors contributing to the change in malaria cases then, and what now? Such comparisons appear to be missing.**

AUTHOR RESPONSE:

This study cites the World Malaria Report and the Global Technical Strategies report, which discuss the malaria mortality rates in Africa. We chose this information because of way they emphasize the comparison of what we hoped would happen versus what has happened. If we reported the prevalence was 63.5/100,000 back in 2015 and 51.8/100,000 in 2025, it would look like we were succeeding in our progress against malaria. So, spot prevalences wouldn’t convey the concern that we share with the WHO. In the past few WHO World Malaria Reports, the authors repeatedly use the phrase “off track”. They use this term to signify that despite going from 63.5 to 51.8 over the past 10 years, we should have been at 15.9/100,000 now. This study wants to convey that lives were lost because we did not or could not use all the tools and information that we had to keep our progress going properly.

This study doesn’t attempt to explain why we were doing well in 2010-2015. The goal of this study is to consider what wasn’t being addressed back in 2012, mainly outdoor sleeping. We believe we had information that back then would have been helpful, but wasn’t being fully addressed. Now that we are further off track and temperatures are rising, considering the aspects of outdoor sleeping may be one of the needed interventions to get things on track again.

**2. To my understanding, Africa already has a hot climate. Global warming does affect change in temperatures (increasing by 1.5-2 degrees Celsius, every year). An important question arises is sleeping outdoors a norm in Africa, or has the habit changed over time? If the habit has evolved overtime, are the average temperatures remarkably different to what were prior to 2012 and after?**

AUTHOR RESPONSE:

We added information regarding the degree of climate change and temperature change in our introduction. We agree with the reviewers’ questions about rates of outdoor sleeping then and now. After this survey was completed and we reviewed the preliminary data, we reexamined the literature regarding outdoor sleeping and malaria prevention. Some citations in this work refer to such studies, but overall, there were very few articles about outdoor sleeping before 2015. Since then, there have been more articles about outdoor sleeping and how it affects malaria prevention. Overall, it is hard to quantify how the rates of outdoor sleeping have changed, however we believe that further research should address this question.

**3. The main focus of the authors appears more on the insecticide treated mosquito nets (ITNs). In 2012, one would expect the ITNs being one of the important measures contributing towards lowering the cases of malaria in the region. Other measures may also be as important to contribute to reductions in malaria cases (especially in 2012), such as insecticide spraying, using mosquito repellants, and covering of exposed parts of the body. Are there any views on how the interviewed village folk protected themselves using other measures? Are these still in practice?**

AUTHOR RESPONSE:

Our survey revealed many practices that people engaged in to fight malaria. Some of these, like keeping the village clean, are only tangentially related to decreasing malaria deaths. Others, like washing nets, might actually be counterproductive. We report this, but do not highlight it.

In this region, it is rare for small children—who are the main victims of malaria—to wear clothing covering the whole body. Changes that have helped to decrease malaria in rich countries, like environmental engineering, air-conditioning and window screens, are not practical in this region. Medical approaches are important. Vaccines may become important. But for primary prevention of infection, ITNs are the major intervention being used, as opposed to insecticide spraying. These are not in any case either/or choices.

The evidence for the life-saving effectiveness and cost-effectiveness of ITNs is impressive. We believe it is appropriate to continue seeking ways to optimize their use.

**4. A question also to ponder is that the use of ITNs is “relatively easy” within households as structures are already in place to hang these nets on the bed or other sleeping places. Are there any views on “erecting” structures so that ITNs could be hanged over sleeping places outdoors?**

AUTHOR RESPONSE:

We continue to work to understand the obstacles and help find solutions to overcome them. In doing so, we have asked the local population for advice on building such outdoor structures for ITN use. For example, we sponsored a contest to develop practical tools for using nets outdoors. The winners received prizes, and we have funded local production and dissemination of a hook made from locally available repurposed bicycle tire tubes and wire (https://youtu.be/OUUquIjP948 ).

**5. The authors have highlighted an important aspect of ITNs; its quality to be durable and long-lasting. From the views of the interviewed population, the ITNs do not appear to be durable. To my understanding WHO has issued guidelines (from 2011) to monitor the durability of ITNs; to withstand about 20 washes, lifespan to be from 1-3 years. Are there any views as to how many times the ITNs had been washed or how long a net had been used by the interviewed population? Is such a practice still being seen in 2023?**

AUTHOR RESPONSE:

The responses did discuss fragility and washing/overwashing but this was not the main focus of this manuscript. It is true that over the last 15 years there have been more studies that show the same ITN fragility concerns as our respondents. We can share separate references from our past literature searches regarding fragility of nets and overwashing upon request.

**6. Conclusions drawn by the authors indicate that the population has a pretty sufficient number of ITNs than what was recommended by WHO. However, this estimate appears more valid for 2012 (and likely contributing to the reductions in malaria cases and deaths). The authors do indicate follow-ups in 2023 also. It will be of much public health importance to compare the perceptions and views of the population of Senegal in 2012 and 2023, which, in turn, will also assist in identifying the factors responsible for malaria resurgence. In view of the ITNs, durability could be explored. One plausible factor for resurgence could be resistance of mosquitoes to the insecticides and repellants. Climate change might also have an impact, however, this need to be explored in greater detail in view of climatic factors such as fluctuations and trends in temperature and precipitation, influencing the rates of malaria and its vector.**

AUTHOR RESPONSE:

It is indeed a multifactorial challenge to prevent malaria, and a combination of all the evidence-based interventions will be required. The authors did not focus on insecticide application or resistance, as there is a separate body of parasitology and entomology research of this. We agree that the perceptions and views of the population must be considered regarding which interventions will be the most appropriate. This is worth further evaluation with research, because if acceptance changes or responses to heat change, public health officials may need to change aspects of interventions.

**Minor edits:
1. Caution needs to be exercised while referencing. For example, in para 1 of the introduction, estimates need to be properly referenced.**

AUTHOR RESPONSE:

We reexamined the source of these estimates, which was the World Malaria Report. In their text and graphs, they do cite another report called the Global Technical Strategy For Malaria 2016–2030. This is a very short document that doesn’t seem to clearly state the data we cite. We do think the Global Technical Strategy document summarizes a lot of data that was responsible for the World Malaria Report’s text. To be sure, we will cite both the World Malaria Report and the Global Technical Strategy report with these important estimates.

**Reviewer 4**

**Data collection is 12 years back.** **How is the results of the study useful in current situation of Malaria in Senegal?**

AUTHOR RESPONSE:

A major impetus for our decision to write this manuscript now, is that if anything, the findings are more urgently relevant now than in 2012 because of global warming.

**- Sentence in lines 63 to 65 need to be rephrased**

AUTHOR RESPONSE:

We agree. This hypothesis statement was reworded.

Originally:

Based on our experiences in villages in Senegal, we hypothesized that we would find that many people in rural areas spend considerable time sleeping outdoors, where it is somewhat cooler than inside their huts.

Now:

The primary hypothesis of this study was that people in this region need more than 0.56 nets per person for adequate protection from malaria-bearing mosquitoes. We further hypothesized that net fragility and outdoor sleeping were major factors contributing to the insufficiency of nets.

**- The rationale of the study is not clear**

AUTHOR RESPONSE:

We added statements clarifying hypothesis and objectives. Details describing these changes can also be seen as our responses to some of the other reviewers’ suggestions above.

**- Paragraph start line 122 can be merged with the paragraph start in line 126 as information seems repeated.**

AUTHOR RESPONSE:

We agree and this has been changed. The information in these two paragraphs both describe the same theme. In one paragraph, we can chronologically narrate the early groundwork of community based participatory research. This starts at pre-survey collaborations. Then we detail all the key points of our process, which we feel are strengths of this survey.

**- Line 172-173 not clear, what does the average population stand for?**

AUTHOR RESPONSE:

This was changed to “The average population per village of all 37 area villages was 235 people.”

**- Discussion part can be more precise
- Conclusion is too long and reiteration of the information already mentioned in Discussion part**

AUTHOR RESPONSE:

Agreed. We have rewritten the Discussion and Conclusion to address these issues.

**- Figure quality poor**

AUTHOR RESPONSE:

We have eliminated the map figure. We have some nice photos in the Supplemental materials. The modified Health Belief Framework figure has been used in other studies and we believe the quality accurately represents how we organized our qualitative data.
